# Atrophic Acne Scar Risk and Reflectance Confocal Microscopy Predictive Features

**DOI:** 10.1111/jocd.70661

**Published:** 2026-04-24

**Authors:** Marco Manfredini, Flavio Fiorito, Tommaso Marchio, Silvana Ciardo, Caterina Longo

**Affiliations:** ^1^ Department of Dermatology, Policlinico di Modena University of Modena and Reggio Emilia Modena Italy; ^2^ Arcispedale Santa Maria Nuova di Reggio Emilia Reggio Emilia Italy

**Keywords:** acne, early treatment, inflammation, risk, scars

## Abstract

**Background:**

Acne scars are considered the result of complex pathophysiologic mechanisms and can have a profound impact on an individual's quality of life, often leading to diminished self‐esteem, depression, and anxiety. Early detection and targeted treatment are crucial. To the best of our knowledge, the correlation between RCM features and acne scar risk has never been assessed.

**Aims:**

Our study aims to explore if any specific morphological RCM feature is associated with acne scar formation and progression.

**Methods:**

Patients were subdivided into groups to compare average values or frequencies of each analyzed variable. To assess the significance of clinical changes between T0 and T1, Student's *t*‐test and Chi‐squared test were performed. Odds ratios were calculated to estimate the relative risk of developing acne scars when a specific parameter was detected.

**Results:**

Our study demonstrates that, in a population predominantly constituted by IGA = 2 grade patients, clinical examination alone does not effectively identify the risk of novel acne scarring. The presence of RCM inflammatory infiltrate was significantly associated with an increased risk of scarring; OR 4.23 (CI: 2.05–8.71).

**Conclusions:**

Identifying specific microscopic parameters, such as vascular changes and inflammatory infiltrate in the skin of acne patients, could be helpful to predict development of new acne scars, allowing for optimized therapies at early disease stages.

## 1 Introduction

The mechanism underlying acne scar formation is not yet fully understood, but the inflammatory process following pilosebaceous unit rupture is thought to play a pivotal role in acne scar development [1, 2]. Although acne severity is an important risk factor for sequelae, the correlation between severity and risk of scarring is not well defined. It is well known that scars can also be induced by mild to moderate acne [3, 4, 5]. Scarring is more common in males, patients with first‐degree relatives that were affected by severe acne, or individuals of African and Asian descent [4, 5, 6].

Acne scars are considered the result of complex pathophysiologic mechanisms, mainly induced by the inflammatory processes that cause the breakdown of normal collagen and elastin fibers, leading to an abnormal fibrotic progression [7, 8]. It was demonstrated that the inflammatory infiltrate of patients prone to scarring is significantly different from patients not prone to scarring. In detail, patients prone to scarring are comparatively characterized by lower CD4+ T cells and more memory/effector skin specific T cells [1, 9].

In the early stages of acne lesions (between 6 and 48 h), the macrophages' recruitment, neoangiogenic process, and vascular adhesion molecules are similar in all patients [7]. However, patients prone to scarring show an increased immune response, especially in the inflammatory resolution phase, with abnormal cellular activation and enhanced macrophages and memory/effector cell recruitment of skin‐homing cell types. Previous studies have shown that *C. acnes* may increase the activity of matrix metalloproteinases (MMPs) 1 and 9, as well as the expression of tissue inhibitors of MMPs (TIMP)‐1 [10]. Additionally, retinoids in their active form constituted by all‐trans retinoic acid (ATRA) are able to downregulate MMP expression while increasing TIMP‐1. This process suggests that retinoids have a protective role against acne scars [10]. Genetic factors such as SELL and TGFB2, play a key role in wound healing and are associated with an increased risk of acne scar development [4, 11, 12]. Abnormal wound healing processes, incorrect remodeling of the extracellular matrix (ECM), and dysregulation of cytokines or growth factors, influence the development of the different types of acne scars [8].

According to recent grading systems, acne scars are atrophic or hypertrophic [1, 2, 13]. Atrophic scars are further subclassified into three types based on their width, depth, and three‐dimensional structure: Ice pick scars, Boxcar scars and Rolling scars. In addition to atrophic scars, this classification also includes hypertrophic scars, papular scars and keloidal scars. The disruption of the ECM can contribute to the formation of various types of acne scars in different ways, inducing atrophic and hypertrophic scars. Acne atrophic scars, the most prevalent type, are characterized by a focal reduction of the ECM that leads to a depressed, pitted, or ice‐pick‐like indentation [1]. This is probably the result of excessive collagen degradation induced by elevated IL‐2, TIMP‐2, and cJUN levels, alongside reduced IL‐10 levels during the early stages of wound healing [9, 14]. From a histopathological standpoint, scars typically display thicker collagen that is stretched and aligned parallel to the epidermis. Hypertrophic scars contain abundant dermal collagen fibers, numerous small blood vessels, and scattered fibroblasts.

Acne scars can have a profound impact on an individual's quality of life, often leading to diminished self‐esteem, depression, and anxiety [11, 12, 14, 15, 16]. Therefore, early detection and targeted treatment are crucial. In vivo reflectance confocal microscopy (RCM) is a non‐invasive technology that allows observation of the epidermis and upper dermis with near‐histologic resolution [17, 18]. RCM, using an 830 nm laser, captures high‐resolution horizontal images of the skin structures, allowing sequential imaging of the same skin area over time [17, 19]. This technology can identify key histopathological features of various types of acne lesions, as well as subclinical changes in seemingly healthy skin of acne patients [17, 18, 20]. Non‐invasive imaging methods are being increasingly utilized for diagnosing, classifying diseases, and assessing treatment outcomes in many skin conditions, including skin cancers and inflammatory skin disorders [21, 22, 23]. The advancement of novel techniques for the non‐invasive microscopic visualization of skin structures has resulted in considerable progress, particularly in the accurate evaluation of acne scars [19, 24]. However, to the best of our knowledge, the correlation between RCM features and acne scar risk has never been assessed. Our study aims to explore if any specific morphological RCM feature is associated with acne scar formation and progression.

## 2 Material and Methods

### 2.1 Population

We conducted a retrospective observational study aimed at evaluating in vivo RCM parameters associated with acne scar formation. The study was conducted in accordance with the ethical principles derived from the Declaration of Helsinki and Good Clinical Practice guidelines, and in compliance with local regulatory requirements guidelines, and in compliance with local regulatory requirements.

The study included patients that underwent a dermatologic visit at the Acne Clinic of the Dermatology Unit at the University of Modena and Reggio Emilia between February 2024 and May 2024. Patient inclusion criteria were: the presence of moderate acne vulgaris (IGA baseline disease severity 2 or 3), and received topical or systemic therapy according to routine clinical practice [25, 26, 27], and had at least 2 complete medical records at baseline (T0) and 5–9 months (T1). Exclusion criteria specified: diagnosis of excoriated acne, positive medical history of severe acne in first degree relatives, any type of physical therapy treatment (laser or peeling) or other aesthetic procedures (including injectables) 3 months prior to T0 up to T1.

A complete medical record included all the following data: demographic information, previous acne treatments, IGA grade, clinical lesion count, clinical scar count, photographic imaging, and RCM imaging.

### 2.2 Scores and Imaging

The clinical assessments included the evaluation of acne severity using IGA [28]. In addition, a detailed clinical lesion count was performed of the total number of acne lesions, categorized into comedones, papules/pustules, and nodules. A key part of the evaluation was the count of facial acne scars, classified by types.

**FIGURE 1:**
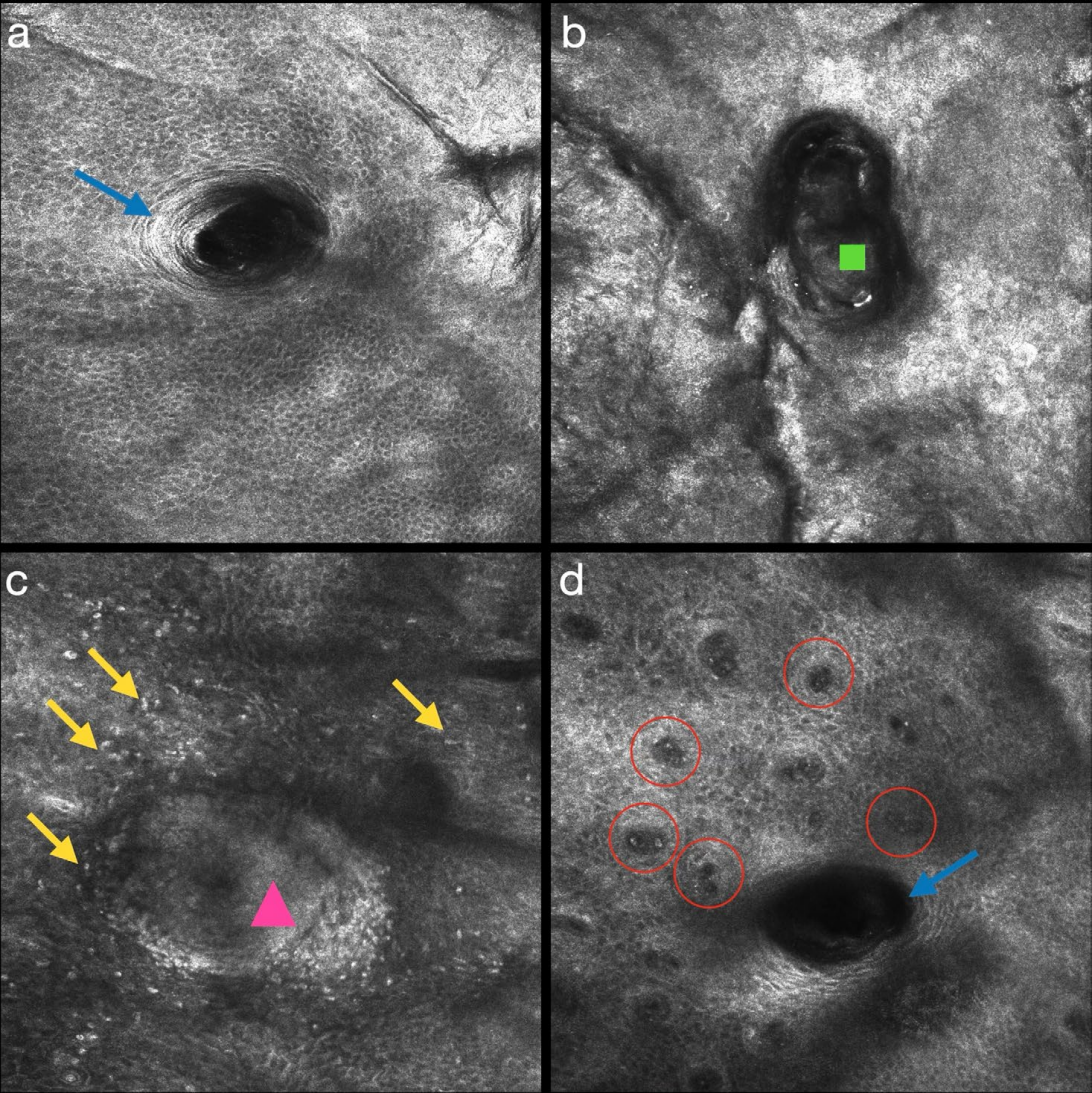
RCM images showing the infundibula of a pilosebaceous unit with thick bright borders (blue arrow) (a); a large and irregular infundibula filled by amorphous material (green square) (b); abundant inflammatory infiltrate composed of many sparse hyper‐reflecting cells (yellow arrow) in the epidermis, coalescing inside the infundibular space (pink triangle) (c), and many dilated vessels (red circles) in the papillae at the periphery of a pilosebaceous unit (blue arrow) (d).

Clinical image acquisition was performed using the VISIA System (Canfield Imaging Systems), and in vivo images with RCM (Vivascope 3000, Vivascope GmbH, Munich, Germany), in accordance with previous studies [17, 29, 30]. A skin target area was selected—the right cheek where clinically evident acne was present for each patient. Three 1 × 1 mm acquisitions were carried out at randomly selected post‐inflammatory erythematous areas in the target area. RCM images were acquired and analyzed according to the already described features (Figure 1) [17, 30, 31].

### 2.3 Statistical Evaluation

The mean (M) and standard deviation (SD) were calculated for continuous variables, while relative frequencies were determined for binary data. For each scar type, patients were subdivided into two main groups according to the change of acne scars over time: G1, when the total number of that type of scar increased over time, and G2, when the total number of that type of scar did not increase over time. In detail the following subgroups were obtained: G1‐rolling and G2‐rolling; G1‐boxcar and G2‐boxcar; and G1‐icepick and G2‐icepick. An analysis of the total number of scars was also performed for each patient, according to the “AllScars” variable, which is the sum of each scar type for each patient. Two additional groups of patients, the G1‐AllScars and the G2‐AllScars, were identified.

To assess the significance of clinical changes between T0 and T1, Student's *t*‐test and Chi‐squared test were performed. Odds ratios were calculated to estimate the relative risk of developing acne scars when a specific parameter was detected. A *p*‐value of less than 0.05 was considered statistically significant. Statistical evaluation was performed with Microsoft Excel (Microsoft Corporation, Redmond, Washington, USA) and R 4.4.0 (R Foundation for Statistical Computing, 2023).

## 3 Results

Twenty‐seven consecutive patients (12 men and 15 women with an average age of 22.2 years) diagnosed with mild‐to‐severe facial acne, undergoing either topical or systemic treatment, were included in the study. Epidemiologic characteristics and previous acne treatment are summarized in Table 1. Clinical and RCM parameters are outlined in Table 2.

**TABLE 1:** Demographic data.

Total number of patients	27
Age, mean + SD	22.19 + 5.51
Gender, male	12
BMI, mean + SD	21.61 + 4.34
Previous acne therapies	
Topical therapies	25 (92.5%)
Tetracyclines	8 (29.6%)
Oral contraceptives	5 (18.5%)
Metformin	3 (11.1%)
Isotretinoin	4 (14.8%)
PDT	2 (7.40%)
Light based therapies	2 (7.40%)

**TABLE 2:** Clinical features and RCM parameters.

	T0	T1	*p*
Clinical features			
IGA	2.11 + 0.32	1.44 + 0.64	0.00
Closed comedos, mean ± SD	9.55 + 6.86	3.88 + 4.47	0.00
Open comedos, mean + SD	3.32 + 3.28	1.48 + 1.61	0.01
Papules and Pustules, mean + SD	14.74 + 10.27	5.72 + 4911	0.00
Inflammatory nodules, mean + SD	0.62 + 1.50	0.24 + 0.66	0.12
Icepick scars, mean + SD	4.48 + 3.86	4.74 + 3.69	0.34
Boxcar scars, mean + SD	2.77 + 2.56	2.92 + 3.18	0.74
Rolling scars, mean + SD	1.48 + 1.52	1.40 + 1.36	0.71
Hyperthrophic scars, mean + SD	0 + 0	0 + 0	na
Total scars, mean + SD	8.74 + 6.31	9.07 + 6.63	0.66
RCM features			
Pilosebaceous units filled with amorphous material, mean + SD	1.43 + 1.14	0.83 (0.89%)	0.00
Pilosebaceous units with bright borders, mean + SD	1.75 + 0.99	0.45 (0.63%)	0.00
Presence of inflammatory infiltrate in the epidermis or upper dermis (%)	38 (46.91%)	14 (17.28%)	0.00
Presence of dilated vessels in the upper dermis (%)	36 (44.44%)	24 (29.62%)	0.07

From a clinical point of view, open comedones and papulo‐pustules significantly reduced over the study period (T0 to T1) (*p* < 0.05); the average number of open comedones was reduced by 55.4% (T0 = 3.3 ± 3.3, T1 = 1.5 ± 1.6), and papulo‐pustules reduced by 61.2% (T0 = 14.7 ± 10.2, T1 = 5.7 ± 4.9).

Eighty‐one RCM acquisitions were analyzed. A significant decrease in the mean number of bright follicles was observed from T0 to T1; the average number of bright follicles decreased by 74.3% (T1 = 1.75 ± 1.0 T1 = 0.45 ± 0.63). At T1, a statistically significant reduction in dilated vessels and inflammatory infiltrate was noted in at least one of the acquisitions, *p* < 0.05.

### 3.1 Acne Scars Predictive Features

**FIGURE 2:**
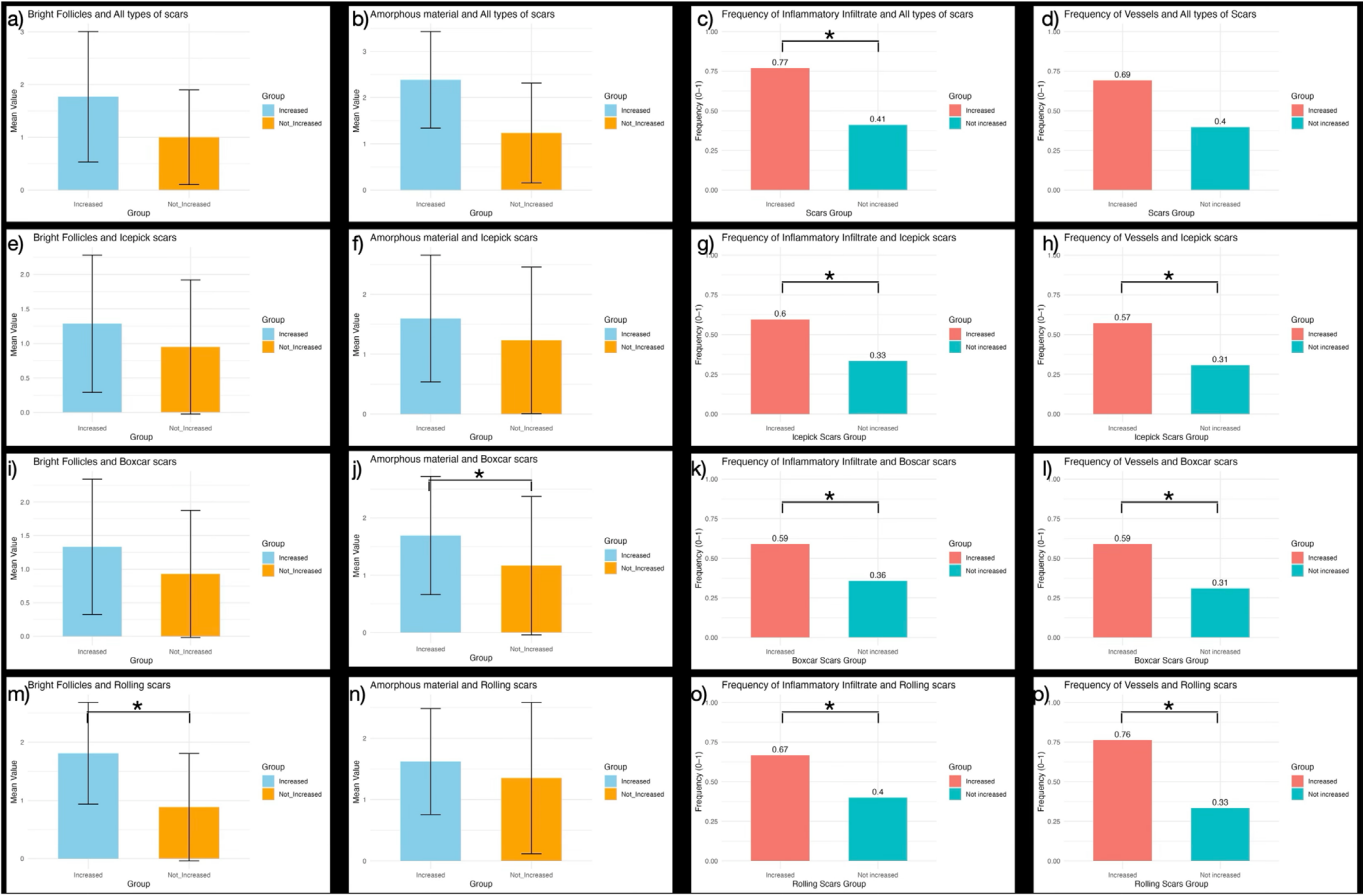
Boxplots representing mean values or relative frequencies of the analyzed parameters among groups, i.e., Patients showing an increase (left boxplot) or not (right boxplot) of the different scar types. Total number of scars was represented by graphs from (a–d). Icepick scars were represented by graphs from (e–h). Boxcar scars were represented by graphs from (i–l). Rolling scars were represented by graphs from (m–p). Error bars are equal to standard deviations. Statistically significant differences among groups are annotated with asterisks (*p* < 0.05).

All the G1 and G2 subgroups were clinically homogeneous; no clinical parameter showed any significant difference among the two groups. RCM features comparisons between G1 and G2 subgroups are described in Figure 2.

#### 3.1.1 All Scars

The odds ratio (OR) of an increase at T1 in the number of all scars, independently of the scar type, was significantly higher if RCM inflammatory infiltrate was detectable at baseline (T0). Therefore, the detection of RCM inflammatory infiltrate was significantly associated with an increase in scars over time compared to its absence, with OR 4.23 (CI: 2.05–8.71).

#### 3.1.2 Icepick Scars

A significant association between icepick scars' increase and the presence of inflammatory infiltrate and dilated vessels was observed, with an OR of 2.9 (CI: 1.19–7.29) and 3.0 (CI: 1.2–7.48), respectively.

#### 3.1.3 Boxcar Scars

The average number of amorphous material at baseline was higher in patients that developed boxcar scars (*p* = 0.03). Similarly, the presence of RCM inflammatory infiltrate and dilated vessels was significantly associated with the development of boxcar scars, with OR of 2.59 (CI: 1.05–6.35) and 3.21 (CI: 1.29–8.0).

#### 3.1.4 Rolling Scars

The average number of bright follicles at baseline was higher in patients who developed rolling scars (*p* < 0.0001). Similarly, the presence of RCM inflammatory infiltrate or dilated vessels was associated with the development of rolling scars with an odds ratio of 3.0 (CI: 1.06–8.52) and 6.4 (CI: 2.05–19.98) respectively.

## 4 Discussion

To the best of our knowledge, this is the first study designed to identify if specific RCM parameters are predictive of acne scarring in patients with acne vulgaris. RCM allows for a rapid and reliable characterization of acne lesions, enabling both morphological and longitudinal evaluations without the need for invasive techniques like skin biopsy, which could leave scars and alter the skin's morphology [18, 30, 32].

Acne scars are likely the most feared complication of the condition, as they are difficult to treat and have a significant impact on patients' quality of life [1, 15, 33].

Our study demonstrates that clinical examination alone is not sufficient to differentiate patients that will or will not develop new acne scars. We also confirm the effectiveness of current acne therapies, routinely prescribed according to current guidelines, with documented reduction in several clinical and RCM acne‐associated parameters [27, 34, 35]. Limitedly to the relatively small number of patients included, the type of therapy administered was not significantly associated with different acne scar development outcomes in the analyzed period.

Our data suggest that the presence of RCM inflammatory infiltrates is significantly associated with a higher risk of atrophic acne scar, independently of the clinical type (icepick, boxcar, or rolling). This reflects the recently demonstrated pathophysiology of the acne scarring process that is induced both by the strength of the inflammatory process of the active acne lesion and by the longer persistence of inflammatory mechanisms during the resolution phase [3, 7]. The abnormal inflammatory process that induces acne scarring may involve the epidermis and the upper dermis, leading to the degradation of collagen and elastin, resulting in a fibrotic process, inducing atrophic lesions [1, 11].

Our findings highlight a strong association between the RCM presence of dilated vessels and acne scar development. Previous studies demonstrated the importance of structural and microvascular changes in scar development, showing that alterations in peripheral blood vessels, both in the dermal papilla and the interfollicular region, may be associated with scarring [24, 30, 36]. Vascular changes are mainly induced by pro‐inflammatory cytokines and act to enhance and amplify the inflammatory cascade and the fibrotic process [1, 8].

Rolling scars that are larger and depressed atrophic scars showed a distinctive association with bright follicle presence [30, 37]. The infundibular changes of several pilosebaceous units demonstrate that a wider area of the epidermis, with respect to icepick and rolling scars, is involved by hyper‐keratinization phenomena, inducing larger inflammatory and fibrotic process [1, 4]. The vicious cycle that involves keratinization phenomena and inflammatory responses is a plausible amplifier that induces deeper scars with depressed and undefined margins [18, 38].

It is important to underline that, for all three types of scars (boxcar scars, icepick scars and rolling scars), clinical parameters were similar at baseline for all patients and did not show the same correlation or predictive power for scar formation as the RCM parameters. This may suggest that RCM could be used for the stratification of acne patients in order to identify subclinical skin changes that are predictive of future scar development [29, 31, 32].

Although these results are promising for the identification of predictive parameters for acne scars development, the study presents some limitations. First, only a small number of patients were analyzed, and a larger dataset is certainly needed for a more robust statistical analysis. Furthermore, given its retrospective nature, the study is subject to biases that could affect the quality and quantity of the data collected. The study power was not designed to define if the different treatments were associated with specific acne scarring outcome.

## 5 Conclusion

Our results demonstrate the importance of RCM assessment in understanding the complex mechanisms of acne scar formation. Identifying specific microscopic parameters, such as vascular changes and inflammatory infiltrate in the skin of acne patients, could be helpful to predict the development of new acne scars, allowing for optimized therapies at early disease stages. Future prospective studies are needed to clarify additional predictive biomarkers of acne scar formation, evolution, and progression.

## Funding

The authors have nothing to report.

## Conflicts of Interest

The authors declare no conflicts of interest.

## Data Availability

The data that support the findings of this study are available on request from the corresponding author. The data are not publicly available due to privacy or ethical restrictions.
